# Pan-European groundwater to atmosphere terrestrial systems climatology from a physically consistent simulation

**DOI:** 10.1038/s41597-019-0328-7

**Published:** 2019-12-16

**Authors:** Carina Furusho-Percot, Klaus Goergen, Carl Hartick, Ketan Kulkarni, Jessica Keune, Stefan Kollet

**Affiliations:** 1 Agrosphere (IBG-3), Research Centre Jülich, 52428 Jülich, Germany; 2 Centre for High-Performance Scientific Computing in Terrestrial Systems, Geoverbund ABC/J, 52428 Jülich, Germany; 3 Jülich Supercomputing Centre, Research Centre Jülich, 52428 Jülich, Germany; 4 Ghent University, Laboratory of Hydrology, Ghent, Belgium

**Keywords:** Natural hazards, Environmental impact, Hydrology

## Abstract

Applying the Terrestrial Systems Modeling Platform, TSMP, this study provides the first simulated long-term (1996–2018), high-resolution (~12.5 km) terrestrial system climatology over Europe, which comprises variables from groundwater across the land surface to the top of the atmosphere (G2A). The data set offers an unprecedented opportunity to test hypotheses related to short- and long-range feedback processes in space and time between the different interacting compartments of the terrestrial system. The physical consistency of simulated states and fluxes in the terrestrial system constitutes the uniqueness of the data set: while most regional climate models (RCMs) have a tendency to simplify the soil moisture and groundwater representation, TSMP explicitly simulates a full 3D soil- and groundwater dynamics, closing the terrestrial water cycle from G2A. As anthopogenic impacts are excluded, the dataset may serve as a near-natural reference for global change simulations including human water use and climate change. The data set is available as netCDF files for the pan-European EURO-CORDEX domain.

## Abstract (summary)

**Table:** 

Measurement(s)	atmospheric water vapour • water-based cloud droplet • evaporation • heat flux • humidity • precipitation process • snowfall • rainwater • pressure of air • radiation • phreatic zone • snow • temperature of air • transpiration • atmospheric wind • atmospheric wind speed • groundwater • Specific Convectively Available Potential Energy • Cape of Mean Surface Layer Parcel • Cloud Ceiling Height • Vertical Integrated Cloud Ice • Total Cloud Fraction • Convective Inhibition of Mean Surface Layer Parcel • Drag Coefficient of Heat • Water Table Depth • Geopotential Height • Height of Boundary Layer
Technology Type(s)	computational modeling technique
Factor Type(s)	Daily
Sample Characteristic - Environment	groundwater • atmosphere • terrestrial biome
Sample Characteristic - Location	Europe

Machine-accessible metadata file describing the reported data: 10.6084/m9.figshare.10355465

## Background & Summary

One of the main impacts of climate change highlighted by the 5th IPCC assessment report (AR5) is “the amplification of temperature extremes by changes in soil moisture”^1–3^, via a positive feedback mechanism that intensifies and increases the frequency of heat waves given the projected increase in summer drying conditions. The associated processes of the terrestrial water and energy cycle result from the interactions between the subsurface, the land surface and the atmosphere. These processes are essential to reproduce, predict and project climatic extreme events in simulations^4,5^. Because in most land surface models (LSMs) water transport and runoff has historically been treated in a simplified way, combined with free drainage lower boundary conditions in the subsurface, soil moisture states and fluxes and interactions between groundwater and soil moisture are biased with multiple impacts especially in areas with shallower groundwater, e.g., on the land-atmosphere coupling and the reproduction of extremes such as heat waves^6^. Recent LSM model improvements for regional climate models (RCMs)^7,8^ can lead to physically consistent interactions between the groundwater, the vadose zone, and the land-surface. Yet, many RCMs and global climate models (GCMs), contributing to CMIP5^3^ or CORDEX^9^ still simplify these interactions. The AR5 acknowledges that the spread in regional climate projections over Europe is still substantial, due to large uncertainties related to natural heterogeneity and chaotic processes, but also due the inherent model structural deficiencies in fully representing two-way, non-linear feedbacks across the terrestrial system.

The dynamic feedbacks between the interacting compartments of the terrestrial system have been studied previously and corroborate the added value in coupling the respective compartment models to improve simulations^7^ and forecasts. Research has focused on the interactions of the soil moisture state and the atmosphere^10^. In addition, the sensitivity of land surface fluxes on the depth of the groundwater table has been demonstrated^11^, particularly for a critical water table depth of 1–5 m where the influence on energy balance is most pronounced, depending on soil heterogeneity and land use type. Similar effects of water table depths on land surface fluxes have been found^5^ using an idealized simulation set-up to infer the effects of topography, land cover, atmospheric forcing and subsurface heterogeneity. A study^12^ on the feedback between groundwater table depth and energy fluxes under changing climate conditions showed that such interactions depend on the prevailing hydrological conditions (energy limited versus moisture limited).

Unlike previous approaches, in which groundwater dynamics are usually not interacting with the atmosphere, terrestrial models such as the Terrestrial Systems Modeling Platform (TSMP) can provide a fully coupled representation of the terrestrial water and energy cycles. The impact of the representation of groundwater in regional climate simulations, has been demonstrated in a number of studies^13,14^ from the catchment to the continental scale. Previous TSMP simulations over Europe concentrated on the 2003 heat wave, showing a significant impact of groundwater states and the related land-atmosphere feedbacks^15^, and demonstrating far reaching impacts of human water use, beyond the local scale through atmospheric moisture transport^16^. However, no physically consistent climatology of the coupled terrestrial hydrologic and energy cycles from the groundwater into the atmosphere is currently available.

In this study, TSMP is run for the European CORDEX domain^17^ as a first step to establish a terrestrial systems climatology for the past decades, with a focus on a physically consistent representation of variably saturated groundwater and overland flow coupled with land surface and atmospheric processes. The dataset we present features daily simulation results since January 1989, but as some grid cells only reach the groundwater equilibrium in 1995, the period applicable for analysis consists of 23 water-years from September 1996 to August 2018 of all essential variables to describe the terrestrial water and energy cycles (Online-only Table 1). The TSMP- G2A data set is a valuable innovative data set to analyze and understand the mechanisms and interactions of water and energy in the terrestrial system including extreme events such as heat waves and droughts.

## Methods

### Data generation method - the terrestrial systems modeling platform (TSMP)

The Terrestrial System Modeling Platform, TSMP^13,18^ version 1.1 is a scale-consistent fully coupled regional Earth system model, comprising the numerical weather prediction model COSMO version 5.01^19^, the land surface model CLM version 3.5^20^ and the surface-subsurface hydrologic model ParFlow^21–23^ version 3.2. The models are externally coupled via the Ocean Atmosphere Sea Ice Soil (OASIS3) Model Coupling Toolkit (MCT) coupler^24^.

The COnsortium for Small Scale MOdelling (COSMO) model system is used by several meteorological services for operational numerical weather prediction (NWP)^19^ and for climate change research as the COSMO Climate Limited area Modelling system (CCLM)^25^. COSMO is a non-hydrostatic limited-area atmospheric model based on the primitive thermo-hydrodynamic Euler equations without scale-dependent approximations, describing fully-compressible flow in a moist atmosphere^19^. In COSMO, both adiabatic transport processes and diabatic processes, such as radiation, turbulence, cloud formation and precipitation are included. The prognostic atmospheric variables in this study encompass pressure, horizontal and vertical wind components, temperature, water vapour, cloud water, cloud ice, rain, snow and the turbulent kinetic energy. At land grid points, additional diagnostic variables, such as the 2 m air temperature and humidity and 10 m wind are provided.

In TSMP, the lower boundary information for COSMO is provided by the widely used Community Land Model (CLM). This boundary condition is composed of surface albedo, upward longwave radiation, sensible heat flux, latent heat flux, water vapor flux, and zonal and meridional surface stresses required by the atmospheric model^20^. These variables are determined by diverse eco-hydrological processes simulated by CLM, such as root water uptake and transpiration by plants. In turn, CLM receives the short- and long-wave radiation, near-surface temperature, barometric pressure, specific humidity, wind speeds, and precipitation from COSMO at each grid point.

In TSMP, the surface water and groundwater flow are calculated by ParFlow. In the coupling, CLM provides the sources and sinks for soil moisture to ParFlow; these are precipitation throughfall and depth-differentiated (root) water uptake from evapotranspiration. In turn, in order to calculate the land surface water and energy balances, CLM receives from ParFlow spatially distributed soil moisture and soil matric potential, which are calculated by ParFlow based on Richards equation and the appropriate initial and boundary conditions in a continuum approach^21,23^. Surface runoff is calculated by a kinematic wave equation in ParFlow^26^. This leads to a dynamic coupling of land surface processes and 3D variably saturated groundwater flow 3D heterogeneity in soil and hydrogeologic hydraulic properties.

The component models are coupled using OASIS3-MCT^13^, following a Multiple Program Multiple Data (MPMD) paradigm in an efficient parallel approach for massively parallel supercomputer environments^18^. Hence all simulations of this study are based on TSMP in fully coupled mode including ParFlow, CLM and COSMO.

### Model setup

**Fig. 1:**
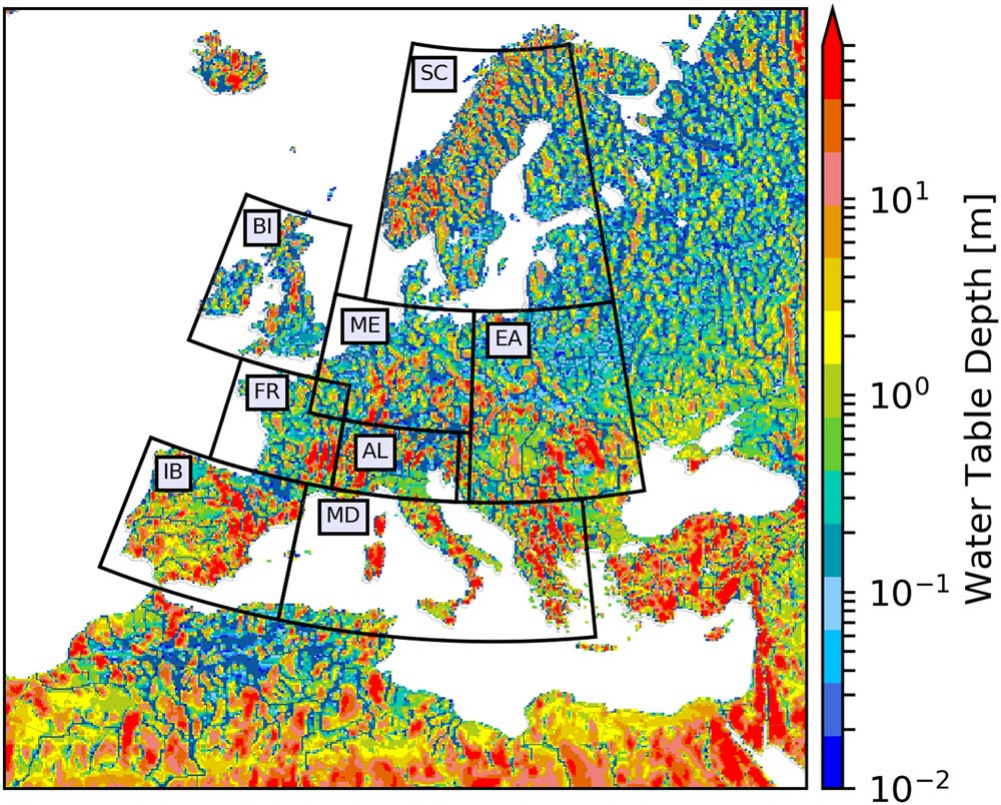
Water table depth [m] climatology from September 1st 1996 to August 31st 2018, represented within the EURO-CORDEX domain (412 × 424 grid cells). The black boxes correspond to the PRUDENCE regions, for which the data time-series validation has been performed.

The model is set up over the European continent (Fig. 1), using a rotated latitude-longitude model grid with a horizontal resolution of 0.11° (12.5 km, termed as the “EUR-11” grid) from the COordinated Regional Downscaling EXperiment (CORDEX) project^17,27,28^, to ensure consistency in comparison with the ensemble of CORDEX RCM experiments (Table 1).

**Table 1:** Model setup of TSMP over the EURO-CORDEX EUR-11 domain.

	ParFlow	CLM	COSMO
Horizontal grid specifications (number of grid points)	436 × 424	436 × 424	444 × 436
Horizontal resolution	0.11°	0.11°	0.11°
Vertical levels	15	10	50
Vertical resolution	variable	variable	variable
Depth/height	57 m	3 m	22 km
Time step	900 s	900 s	60 s

ParFlow and CLM use the same equal-area rotated standardized latitude-longitude grid as COSMO. Please note the grid specification here is including the lateral boundary relaxation zone. The EUR-11 grid mandatory focus area is 424 × 412 grid elements.

In this setup, CLM has 10 soil layers with a total depth of 3 m. These layers correspond to the 10 top layers of ParFlow, which has 5 additional layers with increasing thickness towards the bottom of the model domain reaching a total depth of 57 m to represent most of the active aquifers in Europe^29^. Deeper multi-story confined aquifer system and very deep basin flow at a time scale of hundreds to thousands of years is not accounted for in the model. Along the coastlines the boundary condition is defined as a constant hydraulic pressure with a hydrostatic profile based on a shallow water table of 0.05 m below the land surface. The topography in ParFlow is represented by D4 slopes calculated from the USGS GTOPO30 digital elevation data set, and the terrain following grid transform^30^ with variable vertical discretization in order to improve the simulations for large topographic gradients and coarse lateral resolutions. The time step for ParFlow and CLM is 15 minutes, while COSMO runs with a 60 seconds time step. The coupling frequency between the component models is 15 minutes using averaged values from COSMO.

The hydraulic conductivity parameters for ParFlow are estimated^31^ based on the soil texture taken from the Food and Agricultural Organization (FAO) database^32^. Fifteen different soil types condition the permeability, so that, e.g., grid cells with a soil dominated by clay have a vertical permeability of 0.062 m/hr, while for those composed mainly of sand, a vertical permeability of 0.27 m/hr is defined. The horizontal permeability values are scaled by a factor of 1000, following the scaling effect of the hydraulic parameter according to the grid resolution, resulting from the loss of information of the terrain curvature as consequence of spatial aggregation^33^. The land cover data from the Moderate Resolution Imaging Spectroradiometer (MODIS^34^) data is used to define the plant functional types (PFT) for CLM. The leaf area index, the stem area index, and the monthly bottom and top heights of each PFT are calculated based on the global CLM surface data set^20^. The COSMO model configuration resembles the settings of the CCLM community (https://www.clm-community.eu/).

**Fig. 2:**
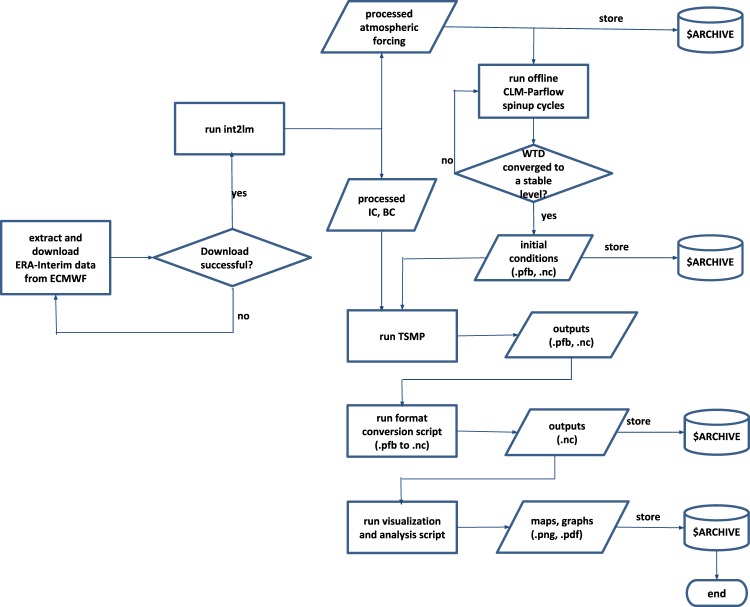
TSMP workflow for climate simulation experiments. Tasks and data flow are presented from the extraction of initial conditions (IC), atmospheric forcing for spin-up and boundary conditions (BC) every 3 h, through pre-processing COSMO inputs with int2lm, model spin-up, climate simulations with TSMP (run TSMP) down to the visualisation and analysis, also showing in which steps input and output data are stored on the Jülich Supercomputing Centre tape archive system ($ARCHIVE).

In order to initialize the model with regard to land surface and subsurface hydrologic and energy states, a dynamic hydrologic equilibrium with the atmosphere must be obtained via a spinup of the model system (Fig. 2, top right boxes). In the spinup, the groundwater-land surface subsystem was simulated using ParFlow-CLM using a 1979–1989 climatologic atmospheric forcing derived from the ERA-Interim^35^ reanalysis. This forcing consists of an annual time series of 6-hourly time steps at each grid point, averaged over 11 years (1979–1989). The reanalysis data were retrieved from the European Centre for Medium-Range Forecasting (ECMWF) MARS archive. The ERA-Interim variables specific humidity, air temperature, 10 m wind speed, precipitation, long and short wave as well as the geopotential height at 0.7° lateral resolution at the lowest ERA-Interim model level were resampled to the EUR-11 grid of TSMP using the COSMO “int2lm” pre-processing software (Fig. 2, top left boxes). A stable dynamic equilibrium with regard to, e.g., soil moisture and groundwater states was achieved after running the ParFlow-CLM model system in a closed loop for 20 cycles. One cycle means one year simulation (January to December) driven by the above mentioned data set. After 20 cycles, the surface and subsurface model states converge and then constitute the initial surface and subsurface conditions for the fully coupled simulation starting from 1989-01-01. The model initialisation in 1989 makes the simulations compatible with the experiment protocol of the EURO-CORDEX RCM ensemble experiments.

The ERA-Interim reanalysis data is also used for the COSMO model atmospheric initial and lateral boundary conditions for the EUR-11 domain throughout the fully coupled model simulations. In order to update the lateral boundaries more frequently than the available 00 and 12 UTC analyses, additional 3, 6, 9 and 15, 18 and 21 UTC forecasts from ERA-Interim were estimated by linear interpolation via the MARS retrieval system of ECMWF to inform the model every 3 h along the boundaries. TSMP is run transient from January 1989 to August 2018, with monthly restarts and no re-initializations of any compartment. Nudging is not used in order to let the evolution of feedback processes evolve freely.

The simulation workflow (Fig. 2) commences with the extraction of initial conditions (IC) and boundary conditions (BC) from ECMWF, followed by the pre-processing of the COSMO inputs with int2lm as a driver for the spin-up runs and as forcing data for the main TSMP simulations. The model spin-up of 20 cycles (years) is performed once before the actual climate simulations with TSMP (*run TSMP* in Fig. 2) can be launched. Concurrently to the model runs, TSMP outputs are continuously post-processed, analysed, visualized and stored at the Jülich Supercomputing Centre following a data-centric simulation and processing paradigm where data moving is kept to a minimum.

The raw outputs from the three component models are archived with 3 h frequency in monthly netCDF files. In addition to data format conversion (binary files from ParFlow to netCDF) and the reduction of the number of files by merging the data in monthly files, post processing also consists of temporal aggregation, calculating daily, monthly and seasonal temporal averages using primarily the Climate Data Operators (CDO, available at http://www.mpimet.mpg.de/cdo). Boundary relaxation zones are removed from each side of the domain. In order to efficiently exchange data and as a means of data provenance tracking, final outputs are transferred to be as much as possible compliant with the CORDEX Archive Design, which in turn is derived from the CMIP specifications. In the process of “CMORization”, data are stored using a predefined Data Reference Syntax (DRS) for the paths and filenames, and defined meta-data per variable as well as global attributes to describe the experiment. This ensures re-usability and interoperability.

## Data Records

The data set is available as netCDF V3 files without compression and is stored at a persistent data repository at the Jülich Supercomputing Centre^36^, as well as at PANGAEA^37^. The spatial resolution and grid specification corresponds to the EURO-CORDEX EUR-11 domain, according to the CORDEX data protocol specification (Version 3.1, 3 March 2014, http://is-enes-data.github.io/cordex_archive_specifications.pdf), with 424 × 412 grid elements on the rotated 0.11° grid. We provide time-series of daily means, aggregated into yearly files. The file names are structured according to the Data Reference Syntax as defined by the EURO-CORDEX archive design: <variable>_<spatial resolution>_<boundary conditions dataset>_<period>_<run identification>_<institute>_<model>_<data version>_<time step>_<initial time step>_<final time step>.

For example, the file clw_EUR-11_ECMWF-ERAINT_evaluation_r1i1p1_FZJ-IBG3-TSMP11_v1_day_20070101-20071231.nc is the vertical integrated cloud ice variable, at the EUR-11 resolution (12.5 km). The run used ECMWF ERA-Interim data, ensemble member r1i1p1, as boundary conditions during the evaluation period, performed at the Research Centre Jülich (FZJ) at IBG-3 Institute, using TSMP version 1.1. It corresponds to the first data-set version (v1), and the file contains daily values between 2007-01-01 and 2007-12-31. Each self-describing file also contains the definition of the geographical coordinate system of the grid (latitudes, longitudes and rotated pole).

### Limitations

**Fig. 3:**
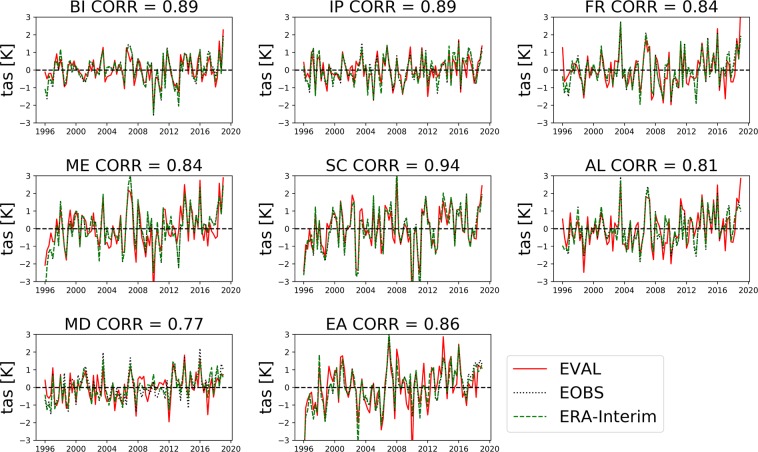
Seasonal anomalies of the simulated (EVAL) 2 m air temperature compared to the E-OBS v19 dataset averaged over each PRUDENCE region. The gray line represents E-OBS v19 dataset whereas the continuous red line represents TSMP simulated data and the green dashed line, ERA-Interim data. The corresponding sample covariance or cross-correlation (CORR) values with no lag time follow the PRUDENCE region code (BI - British Isles, IB - Iberian Peninsula, FR - France, ME - Mid Europe, SC - Scandinavia, AL - Alps, MD - Mediterranean and EA - Eastern Europe, see Fig. 1).

**Fig. 4:**
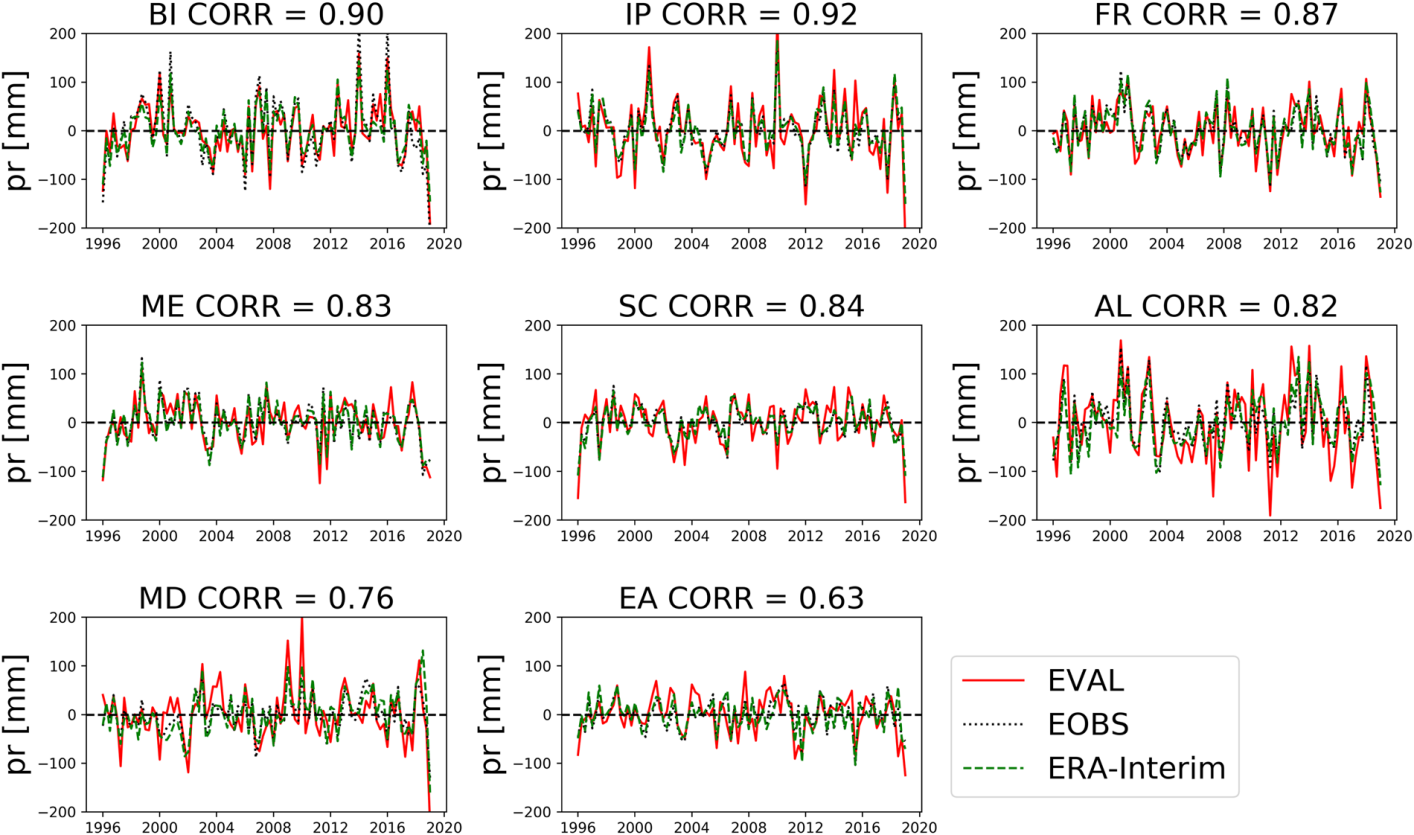
Seasonal anomalies of the simulated (EVAL) precipitation (mm) compared to the E-OBS v19 dataset and ERA-Interim over each PRUDENCE region. As in Fig. 3, only for precipitation instead of temperature data.

Due to the fact that the simulation was run transient after initialisation in 1989-01-01, without re-initializations, the model solution is expected to diverge from the forcing data as well as observations at the event scale. However, the regional anomalies compared to reference observational datasets show that the model captures the system dynamics and succession of dry/wet years, as well as heat waves and cold spells (Figs. 3 and 4). Furthermore, the 12.5 km resolution is not high enough to explicitly resolve convection and the development of convective precipitation^38,39^ or the hydrology of smaller headwater catchments, including flash-flood prone watersheds.

## Technical Validation

The current experiment was designed to produce a near-natural climatology of the physical states of the terrestrial system, without the influence of, e.g., human water use. Accordingly, no real-world measurements are available for this type of system state for comparison and validation. Nevertheless, Figs. 3 and 4 show that the model reproduces the succession of warm/cold and wet/dry seasons on the regional scale for the PRUDENCE analysis regions^40^ (boxes in Fig. 1), compared to the one of the commonly used reference datasets for temperature and precipitation, the 0.25 degrees gridded European Climate Assessment and Dataset^41^ (E-OBS v19, ECA&D). The Pearson’s correlation values between the simulated and observed data series show good agreement in most European regions, with scores ranging from 0.73 to 0.94 for mean temperature anomalies and from 0.62 to 0.88 for precipitation anomalies. The time-series used for the validation period comprises 22 years from 01-01-1996 to 31-12-2018, because the model had not reached full groundwater states equilibrium in all grid cells until 1995.

**Fig. 5:**
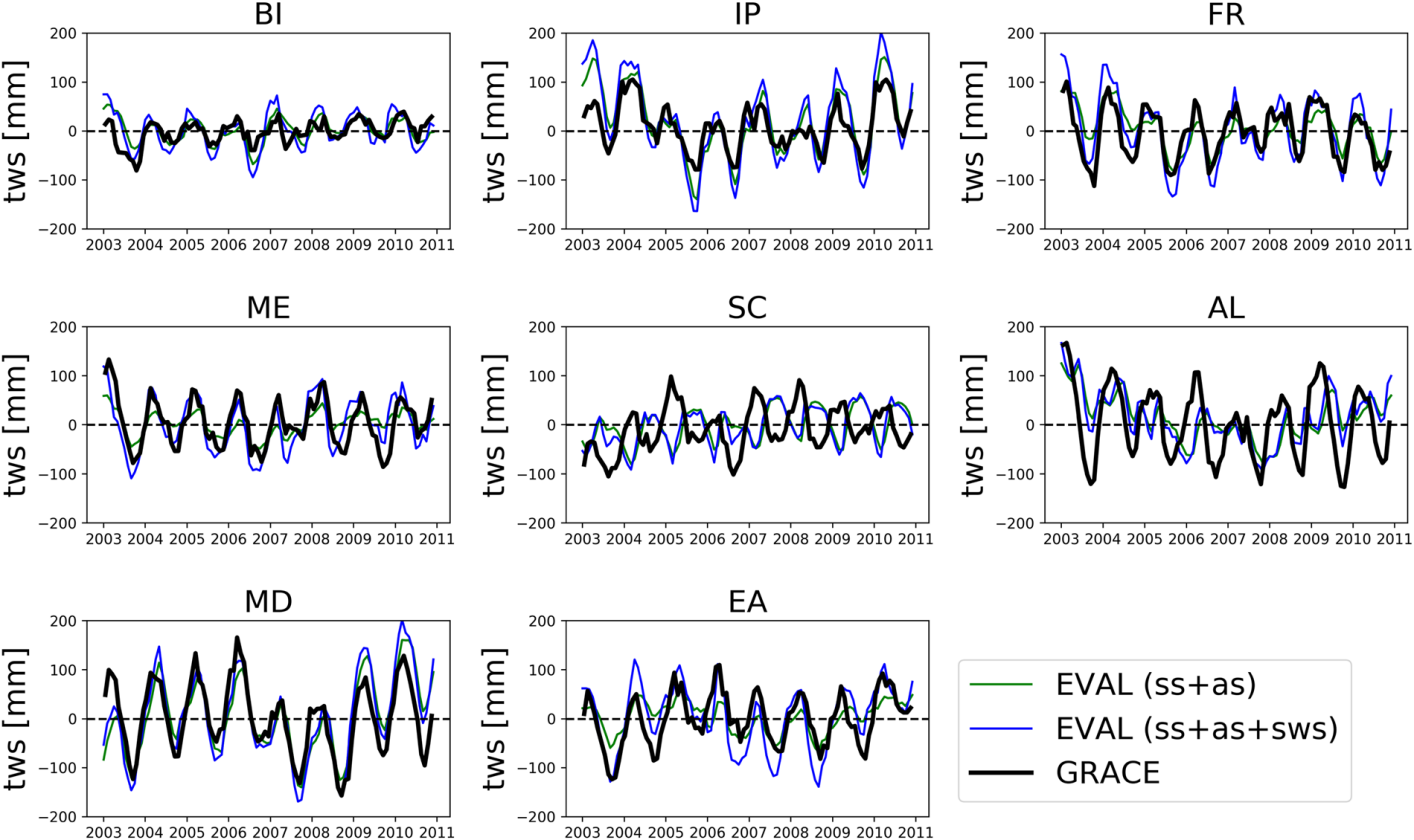
Storage anomalies s calculated for the entire soil column including soil water, ss, and aquifer, as storages (ss + as) and surface water storage (ss + as + sws) on the time period used on the GRACE mascon data set, 2003–2011, over each PRUDENCE region (Fig. 1).

The total column water storage simulated by TSMP was assessed by comparing to the observations of GRACE^42^ (Fig. 5). The total column water storage i, j (L) from the land surface to the bottom of the aquifer constitutes an integrated measure of water resources calculated as follows^43^ in Eq. (1):

1 $${s}_{i,j}=\mathop{\sum }\limits_{k}^{nz}{sat}_{i,j,k}{por}_{i,j,k}d{z}_{k}$$

where sat_i,j,k_ is the relative saturation (−), por_i,j,k_ the porosity (−) for a pixel with indices i, j, k in the lateral and vertical direction, respectively, dz_k_ is the extent of a vertical grid cell (L) and nz is the number of grid cells in the vertical direction. Monthly anomalies were calculated for each pixel and then within each PRUDENCE region. Figure 5 shows that the column water storage simulated by TSMP is in good agreement with the GRACE data in most PRUDENCE regions, but there are discrepancies in the Alpine region (AL) as well as in Scandinavia (SC).

The model capability of reproducing water storage and water table depth have been discussed in previous studies focusing on the European heat wave of 2003^15,16^. The overall WTDs simulated with TSMP are comparable to the WTD composition by an observation based global gridded model of WTD^44^ with large-scale patterns, following the terrain, representing a shallow WTD along the coastlines and in arid valleys and also inundated wetlands in lowland regions, e.g., Netherlands^15^.

## Usage Notes

As most climate data in netCDF, starting with a quick visualization can be easily achieved with any netCDF viewer such as ncview (http://meteora.ucsd.edu/~pierce/ncview_home_page.html), meta data of the file may be best viewed using the ncdump command; a list of software tools and libraries for using netCDF is available from the developers of the netCDF format at UCAR (https://www.unidata.ucar.edu/software/netcdf/software.html). The CDO software is a collection of operators for standard processing of climate model data and can be directly used to work with this dataset taking into account the spatial reference of the data as well as the temporal information. The application is straightforward and allows a wide range of calculations from space-time aggregation to sophisticated climate index calculations. For more personalized analysis and visualization, we also recommend using Python with specific libraries such as Pandas or xarray. Codes created specifically for post-processing TSMP are also available with the TSMP release version.

## Data Availability

Stable release versions of TSMP are provided through a git development repository available at the model’s website (https://www.terrsysmp.org). The release version includes extensive instructions for installing the system, including sample reference test cases for typical application examples, as well as a suite of pre-processing and post-processing tools. TSMP is essentially released without its component models, i.e., the release contains the built system, all configuration files, such as namelists, for the sample cases, the component model code patches and all coupler related modifications. The user must download the component models from their respective separate repositories: All ParFlow releases are available via GitHub (https://github.com/parflow/parflow). The official CLM website (http://www.cgd.ucar.edu/tss/clm/distribution/clm3.5/index.html) offers all links to documentation, source code, and input data for the stand-alone version release of CLM as used in this study. The COSMO model is available only after registration (cosmo-licence@cosmo-model.org) and is also free of charge for research applications. More information on the procedure and licensing terms are available at the COSMO model website (http://www.cosmo-model.org/content/support/software/default.htm). It must be noted that the TSMP model system supports various combinations of different component model versions; e.g. ParFlow only, ParFlow-CLM, CLM-COSMO, ParFlow-CLM-COSMO.

## Online-only Table

**Online-only Table 1:** TSMP EUR-11 variable outputs.

Variable names	Long names	Units	Model	Levels	Size (G)
awt*	Atmosphere Total Water Content	kg m-2	COSMO	1	7,1
capec*	Specific Convectively Available Potential Energy	J kg-1	COSMO	1	7,1
capeml*	Cape of Mean Surface Layer Parcel	J kg-1	COSMO	1	7,1
ceiling*	Cloud Ceiling Height (Above MSL)	m	COSMO	1	7,1
cli*	Vertical Integrated Cloud Ice	kg m-2	COSMO	1	7,1
clt	Total Cloud Fraction	—	COSMO	1	7,1
clw*	Vertical Integrated Cloud Water	kg m-2	COSMO	1	7,1
evspsbl	Evaporation	mm s-1	CLM	1	7,1
hfls	Surface Upward Latent Heat Flux	W m-2	CLM	1	7,1
hfss	Surface Upward Sensible Heat Flux	W m-2	CLM	1	7,1
hudiv*	Atmosphere Water Divergence	kg m-2	COSMO	1	7,1
hur2*	2 m Relative Humidity	%	COSMO	1	7,1
hur200(500,850)*	Relative Humidity (at 200, 500 and 850 hPa)	%	COSMO	3	7,1 (x3)
hus2*	2 m Specific Humidity	kg kg-1	COSMO	1	7,1
hus200(500,850)	Specific Humidity (at 200, 500 and 850 hPa)	—	COSMO	3	7,1 (x3)
incml*	Convective Inhibition of Mean Surface Layer Parcel	J kg-1	COSMO	1	7,1
pgw*	Groundwater Pressure	m H2O	PF	15	216
pr	Precipitation	kg m-2	COSMO	1	7,1
prc	Convective Precipitation	kg m-2	COSMO	1	7,1
prg*	Large Scale Precipitation	kg m-2	COSMO	1	7,1
prsn	Snowfall Flux	kg m-2 s-1	CLM	1	7,1
prso*	Precipitation on Ground	kg m-2 s-2	CLM	1	7,2
prt*	Total Rain Water Content Vertically Integrated	kg m-2	COSMO	1	7,1
ps	Surface Air Pressure	Pa	COSMO	1	7,1
psl	Sea Level Pressure	Pa	COSMO	1	7,1
rlds	Surface Downwelling Longwave Radiation	W m-2	CLM	1	7,1
sgw*	Groundwater Saturation	—	PF	15	215
snt*	Total Snow Content Vertically Integrated	kg m-2	COSMO	1	7,1
ta200(500,850)	Air Temperature (at 200, 500 and 850 hPa)	K	COSMO	3	6,9 (x3)
tas	Near-Surface Air Temperature	K	CLM	1	7,1
tch	Drag Coefficient of Heat	—	COSMO	1	7,1
td2	2 m Dew Point Temperature	K	COSMO	1	7,1
trspsbl*	Transpiration	W m-2	CLM	1	7,1
ua200(500,850)	Eastward Wind (at 200, 500 and 850 hPa)	m s-1	COSMO	3	7,1 (x3)
uas	Eastward Near-Surface Wind Velocity	m s-1	COSMO	1	7,1
va200(500,850)	Northward Wind (at 200, 500 and 850 hPa)	m s-1	COSMO	3	7,1 (x3)
vas	Northward Near-Surface Wind Velocity	m s-1	COSMO	1	7,1
wtd*	Water Table Depth	m	PF	1	15
zg200(500,850)	Geopotential Height (at 200, 500 and 850 hPa)	m	COSMO	3	7,1 (x3)
zmla	Height of Boundary Layer	m	COSMO	1	7,1
